# A novel low –cost and sensitive sensor for the voltammetric nano detection of linezolid antibiotic in real samples using carbon paste electrode modified with calcium oxide nanoparticle combined with electropolymerized D-alanine

**DOI:** 10.1186/s13065-025-01663-3

**Published:** 2025-11-15

**Authors:** Mahmoud Khodari, Nahla Z. Hassan, Adila E. Mohamed, Mohamed N. Rashed

**Affiliations:** 1 Chemistry department, Faculty of Science, South Valley University, Qena, Egypt; 2 Chemistry department, Faculty of Science, Aswan University, Aswan, Egypt

**Keywords:** Carbon paste electrode, Eggshell, Linezolid, Voltammetry, Nanoparticles, D-alanine

## Abstract

This study reports the development of a novel, low-cost, and highly sensitive sensor for the rapid and efficient electrochemical detection of trace amounts of the antibiotic Linezolid (LNZO) in both synthetic solutions and real samples. The proposed sensor is based on a carbon paste electrode (CPE) modified with calcium oxide nanoparticles (CaO-NPs) and further enhanced through electropolymerization of D-alanine (PoDA). Eggshell waste was recycled as a low-cost and eco-friendly source of calcium oxide nanoparticles (CaO-NPs). The synergistic effect of CaO-NPs and the electropolymerized D-alanine (PoDA) film enhanced the electrocatalytic activity of the carbon paste electrode (CPE) toward Linezolid (LNZO). The fabricated sensor was characterized using FTIR, XRD, and SEM techniques. It exhibited excellent electro-oxidation performance toward LNZO, demonstrating high sensitivity, a very low detection limit, good selectivity, and acceptable reproducibility. Differential pulse voltammetry provided a wide linear range (0.005–0.1 µM) with a detection limit of 1.27 nM. Importantly, the sensor successfully detected LNZO in real samples, achieving recovery rates between 95.70% and 100.20%, confirming its effectiveness for practical applications the developed sensor confirmed the ability to identify LNZO in real samples, yielding acceptable recovery percentages within the range of 95.70% to 100.20%, proving the method’s effectiveness in practical applications.

**Supplementary Information:**

The online version contains supplementary material available at 10.1186/s13065-025-01663-3.

## Background

**Fig. 1:**
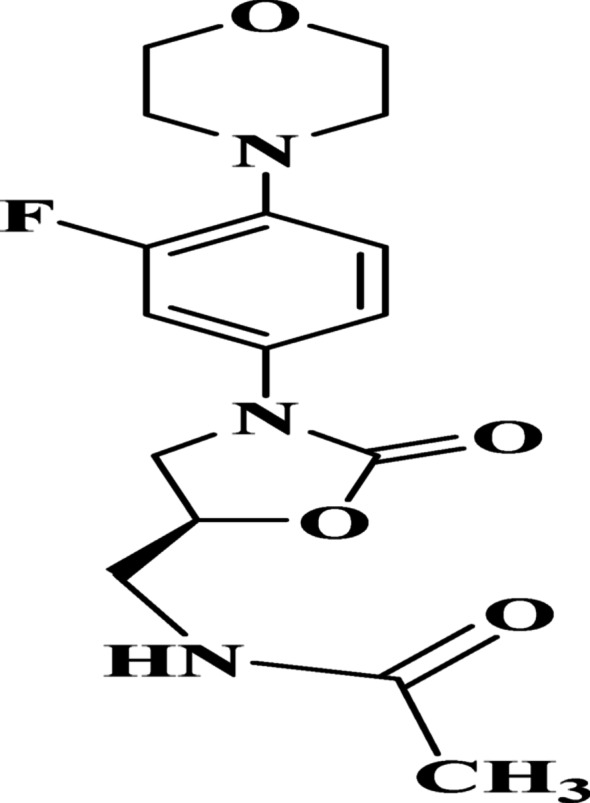
Molecular structure of Linezolid antibiotic

Linezolid (LNZO), chemically designated as (S)-N-(3-[3-fluoro-4-(morpholin-4-yl) phenyl]-2-oxo-1, 3-oxazolidine-5-ylmethyl) acetamide (Fig. 1), is the first commercially available oxazolidinone antibacterial agent to reach clinical application. It is widely employed for the treatment of multidrug-resistant Gram-positive bacterial infections and acts by inhibiting bacterial protein synthesis through a unique mechanism. Despite its therapeutic efficacy, prolonged administration of LNZO has been associated with adverse effects, particularly peripheral neuropathy, underscoring the need for strict monitoring to ensure patient safety.

A variety of analytical techniques have been developed for the determination of LNZO, including UHPLC-DAD [1], spectrophotometric and spectrofluorometric methods [2, 3], HPLC-UV [4–6], and LC-MS/MS [7–9]. However, many of these techniques are labor-intensive, require expensive instrumentation, and involve lengthy sample preparation. To address these limitations, significant efforts have been devoted to developing alternative analytical approaches, particularly electroanalytical methods, for the quantitative determination of LNZO. Electroanalytical techniques are increasingly favored in pharmaceutical analysis due to their distinct advantages, including availability, low cost, sensitivity, selectivity, and accuracy [10–16]. As a result, it’s vital to enhance a simple, cheap, and sensitive detector that executes the voltametric method.

Several electrochemical methods for the determination of LNZO have been reported in the literature [17]. developed a voltammetric method for the analysis of LNZO in pharmaceuticals and human plasma using a glassy carbon electrode (GCE) [18]. investigated the electrochemical behavior of LNZO at both carbon paste electrodes (CPE) and pencil graphite electrodes (PGE), demonstrating their applicability in human urine and pharmaceutical formulations [19]. proposed an electrochemical sensing platform based on a bromocresol green-modified carbon paste electrode with multiwalled carbon nanotubes (MWCNT/BCG/CPE) for LNZO determination. Similarly, Others [20] used a GCE modified with electro-reduced graphene oxide–bentonite sodium composite (ERGO-BEN-GCE) to analyze LNZO in bulk drugs, tablets formation, and urine samples. Ali K. Attia et al. [21] developed a voltammetric sensor using modified CPE with multiwalled carbon nanotubes (MWCNTs) for the determination of LNZO, either alone or in combination with meropenem (MERO) and theophylline (THEO) in plasma samples. Nada F. Atta et al. [22] employed a modified GC sensor for the simultaneous determination of LNZO and meropenemME in human serum. The GC was modified with a thin layer of graphene (Gr), followed by casting two consecutive thin layers of carbon nanotubes-hydroquinone mixture (CNT-HQ) and Fe-Ni alloy nanoparticles, enabling sensitive voltammetric detection. Daniele Merli et al. [23] also utilized GCE for the quantification of LNZO in pharmaceutical formulations using DPV. Overall, both glassy carbon and carbon paste electrodes have been widely investigated as working electrodes for LNZO determination, often in combination with surface modifications to enhance sensitivity, selectivity, and applicability across pharmaceutical and biological matrices.

The widespread application of CPEs in electroanalytical studies can be attributed to their facile modification, which allows tailoring of their properties to enhance both selectivity and sensitivity. CPE are considered ideal electrodes for the determination of a wide range of analytes owing to its low cost, renewable surface, and simplicity of modification with various substances that enable the enhancement of selectivity and sensitivity by increasing its activated surface area [24]. In particular, the incorporation of metal oxide nanoparticles onto CPE surfaces has been extensively explored, as these nanomaterials significantly enhance electrocatalytic activity and facilitate electron transfer within redox systems [25, 26].

Calcium oxide nanoparticles (CaO-NP) have emerged as promising surface modifiers for CPE. n this study, eggshell waste was utilized as a low-cost precursor for CaO-NP synthesis, as it consists predominantly of CaCO₃ (>97%) [27]. The resulting CaO-NPs possess several advantageous properties, including low toxicity, high adsorption capacity, excellent catalytic activity, and economic viability. These features have enabled their widespread application as industrial catalysts, adsorbents in water treatment, sorbents for CO₂ capture, and purifiers of exhaust gases. Their multifunctional characteristics make CaO-NPs particularly attractive for integration into electrochemical sensing platforms [28].

The electro-polymerization of D-alanine to form poly-D-alanine (PoDA) has been reported to generate a polymeric film enriched with amine and carbonyl functional groups, which can interact with target analytes through hydrogen bonding and/or electrostatic interaction. This functional layer promotes analyte accumulation at the electrode surface, thereby enhancing the electrode’s sensitivity [27–29]. Hence, by considering all aforementioned merits of CPE, CaO-NPs, and PoDA, the PoDA@CaO-NPs/CPE was fabricated in this study for the electrochemical detection of LNZO.

In the present work, a novel, cheap, simple, and sensitive sensor based on PoDA@CaO-NPs/CPE for the determination of LNZO was developed. The modified electrode exhibited a venerable response toward LNZO compared to bare CPE. Under optimized conditions, the fabricated sensor was effectively functionalized for sensing of LNZO in commercial pharmaceutical tablets and serum samples.

## Materials and methods

### Chemicals and reagents

All materials used in this study were employed without further purification. D-alanine and the pure Linezolid (LNZO) reference standard (≥ 98%) were purchased from Sigma-Aldrich. Pharmaceutical Averozolid tablets were obtained from a local pharmacy. A stock solution of LNZO (1.0 × 10⁻³ M) was freshly prepared by dissolving 33.73 mg of the drug in 100 mL of methanol and stored at 4 °C. Working solutions were obtained by serial dilution of the stock solution with methanol.

### Apparatus

A potentiostat (VersaSTAT 4) was used to perform all voltammetric measurements. The electrochemical system consisted of three electrodes: PoDA@CaO-NPs/CPE as the working electrode, Ag/AgCl as the reference electrode, and platinum wire as the counter electrode. FT-IR spectra were recorded using a PerkinElmer spectrometer, while XRD patterns were obtained at 25 °C with a Bruker D8 Advance (Germany). The surface morphology of the modified electrodes was examined using a JEOL JSM-5500 LV scanning electron microscope (Japan) .

### Manufacture of used working electrodes

The bare carbon paste electrode (BCPE) was fabricated as described in our previous work [29]. Calcium oxide nanoparticles (CaO-NPs) were synthesized according to an earlier report [30]. A mixture of graphite powder and CaO-NPs was prepared by grinding in an agate mortar at a weight ratio of 60:15 (w/w %), followed by the addition of 0.25 g of molten paraffin wax. The components were thoroughly blended to obtain a homogeneous paste, which was then packed into an insulin syringe, similar to the preparation of BCPE.

The surfaces of the modified electrodes were activated by cyclic voltammetry (CV) within the potential window of − 1.8 V to + 1.5 V in phosphate buffer solution (PBS) until stable voltammograms were obtained. Electropolymerization of D-alanine was carried out on CPE and CaO-NPs/CPE by immersing the electrodes in a 1.12 mM D-alanine solution prepared in PBS (pH 7.4). CV was applied to produce PoDA/CPE and PoDA@CaO-NPs/CPE by performing successive scans in the potential range of − 0.6 V to + 2.0 V at a scan rate of 100 mV/s. After polymerization, the electrodes were thoroughly rinsed with double-distilled water to remove any physically adsorbed species .

### Real samples Preparation

Human serum samples were collected from healthy volunteers (over 21 years old) at South Valley University Hospital. The research protocol was conducted in accordance with the South Valley University Research Code of Ethics (SUV-ٌٌٌRُُEC). Serum samples were prepared as described in our previous work [29] using a dilution factor of 10.

Pharmaceutical Averozolid™ tablets (600 mg) were finely ground in an agate mortar with a pestle. An appropriate amount of the powder was dissolved in methanol and sonicated for 30 min. The resulting solution was filtered, and the residue was washed three times with methanol. The filtrate was then diluted with the same solvent to obtain the desired concentration. For electrochemical measurements, the required concentrations were prepared by spiking specific volumes of the solution into the electrochemical cell and diluting with phosphate buffer solution (PBS, pH 3.0).

## Results and discussion

### PoDA coating on CaO-NPs/CPE via electro-polymerization

**Fig. 2:**
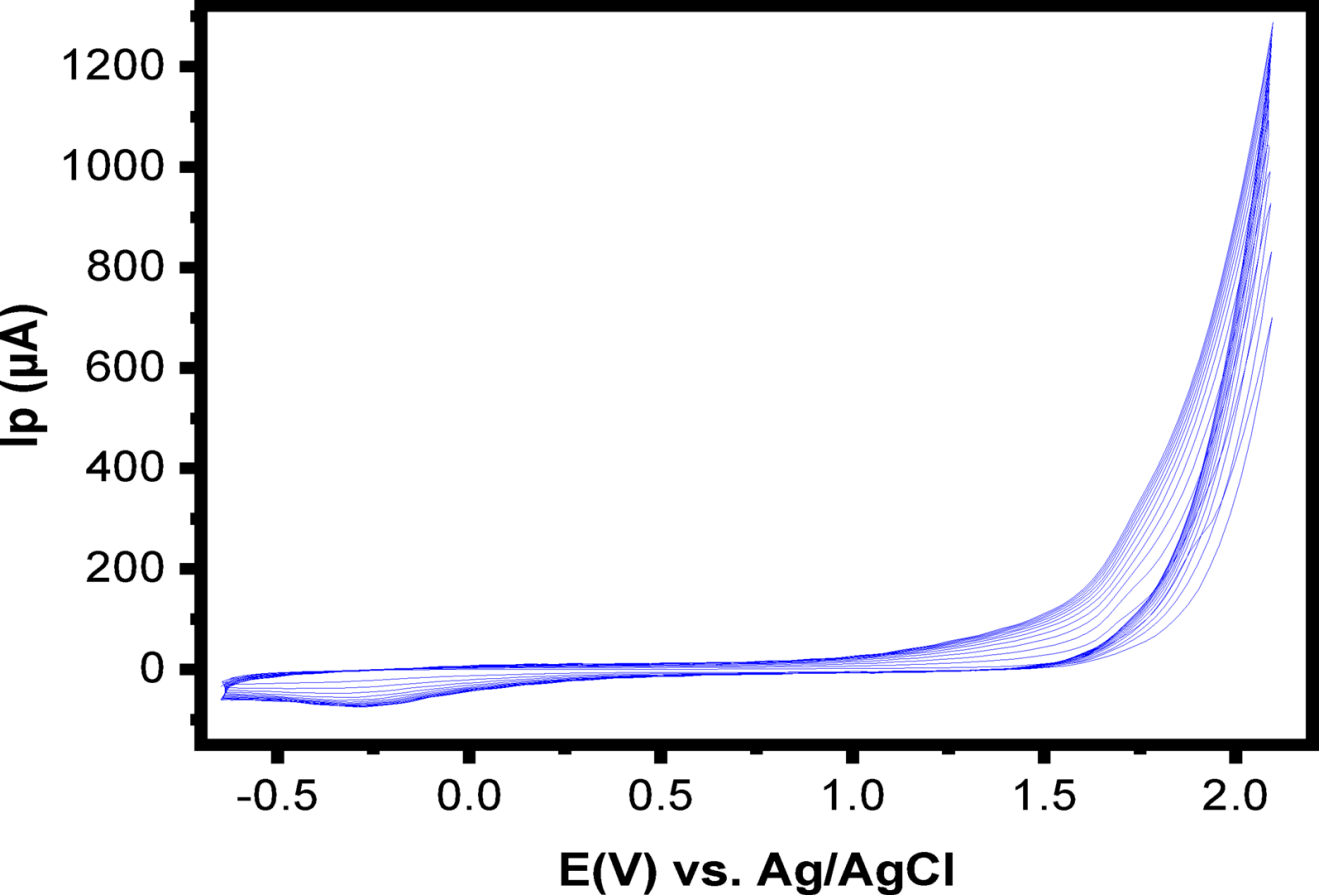
CVs for the electro-polymerization of D-alanine (1.12 mM) in PBS (pH = 7.4) at CaO-NPs/CPE, (number of cycles = 13 cycles)

Coating the surface of CaO-NPs/CPE with a PoDA layer via electropolymerization significantly enhanced its electroanalytical performance. The PoDA film was formed by applying consecutive cyclic voltammetry (CV) scans at a rate of 100 mV/s in a 1.12 mM D-alanine solution prepared in PBS (pH 7.4), within a potential window of − 0.6 to + 2.0 V. As shown in Fig. 2, the redox peak currents increased with successive potential cycles, indicating progressive film formation. This behavior suggests that the transformation of the D-alanine monomer into a polymeric PoDA film improved the electrocatalytic activity of the electrode [31].

### Morphological characterization of CaO-NPs

**Fig. 3:**
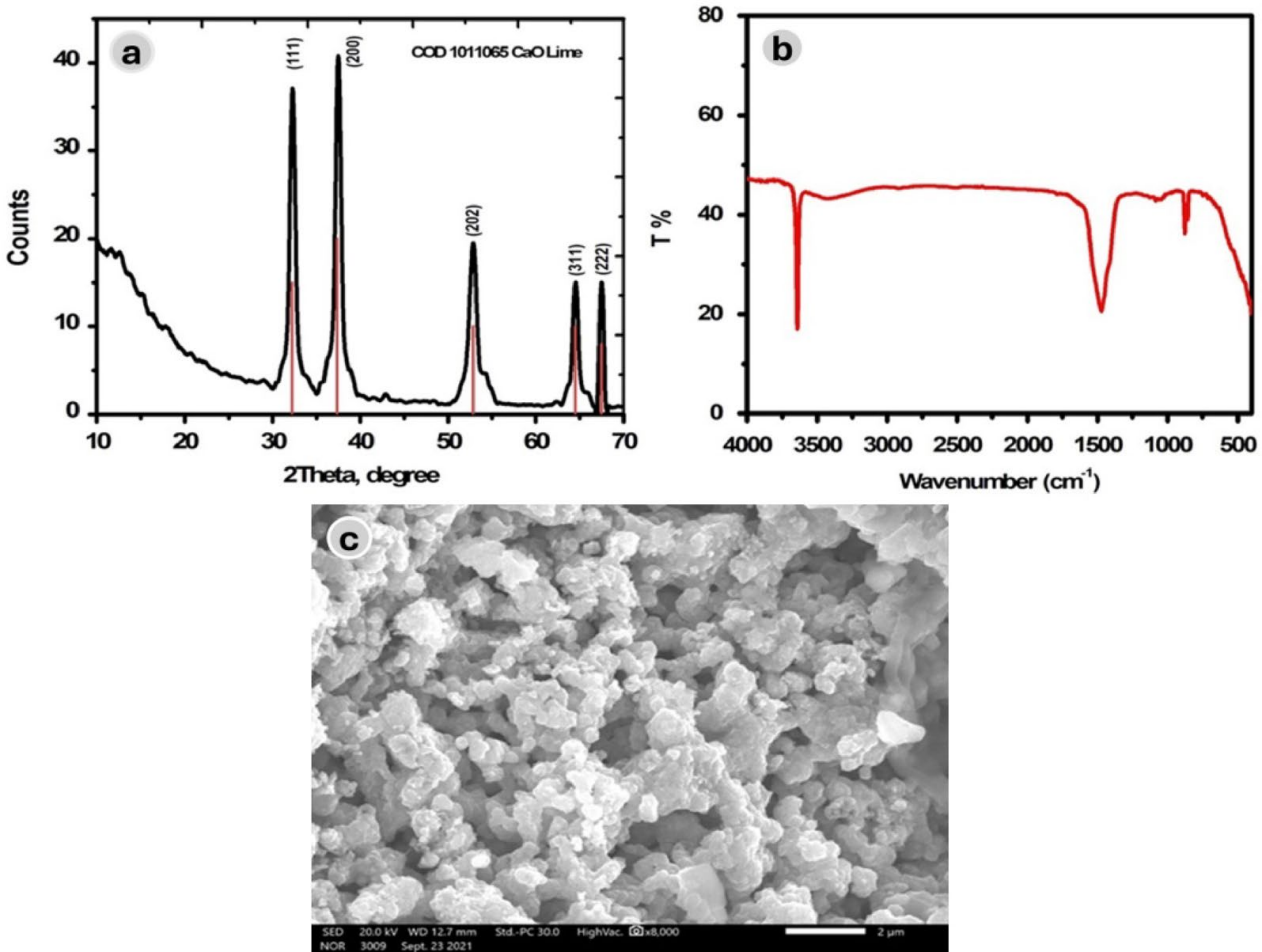
**a** XRD pattern of CaO-NPs, **b** FTIR spectrum of the prepared CaO-NPs, and **c** SEM image of CaO-NPs

The synthesized CaO-NPs were characterized using XRD, FTIR, and SEM techniques. The structural and crystallographic features were further analyzed by X-ray diffraction (XRD), as shown in Fig. 3A. The XRD pattern displayed sharp and intense diffraction peaks at 2θ values of 32.24°, 37.40°, 53.93°, 64.24°, and 67.47°, corresponding to the (111), (200), (202), (311), and (222) crystal planes, respectively. These reflections are consistent with the standard cubic phase of CaO (COD 1011095, CaO Lime), confirming the high crystallinity of the nanoparticles. The absence of any additional peaks indicates phase purity. The average crystallite size, calculated using the Debye–Scherrer equation [32], was estimated to be approximately 25 nm .

The high purity of the synthesized CaO nanoparticles (CaO-NPs) was further verified by FT-IR spectroscopy (Fig. 3B). Characteristic Ca–O stretching vibrations were observed at 858 and 875 cm⁻¹, confirming the successful formation of CaO-NPs [33]. In addition, broad absorption bands at 3642 and 1472 cm⁻¹ were attributed to hydroxyl groups arising from adsorbed water molecules on the nanoparticle surface [28].

The morphology and surface characteristics of the CaO-NPs were examined using scanning electron microscopy (SEM) (Fig. 3C). The SEM images showed that the nanoparticles were predominantly spherical, nanosized, and uniformly distributed. Although some aggregation was observed, the particles retained a porous and homogeneous texture, consistent with the typical morphology of CaO nanoparticles .

### 3.3 morphological characterization of the prepared working electrodes via SEM

**Fig. 4:**
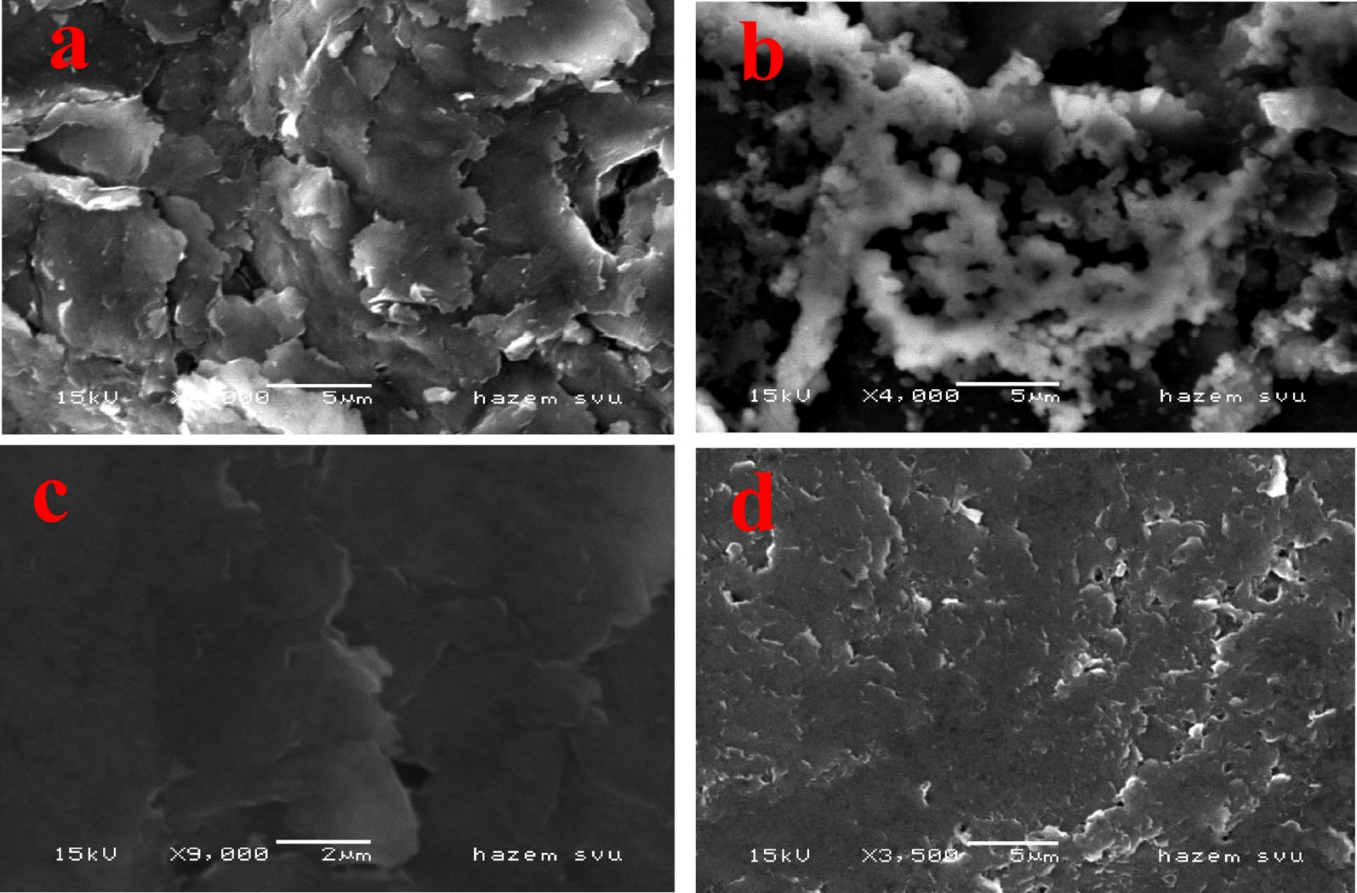
SEM micrograph of (**a**) BCPE, **b** CaO-NPs/CPE, **c** PoDA/CPE, and **d** PoDA@CaO-NPs/CPE

The surface morphology of BCPE is shown in Fig. 4a, where micrometer-sized graphite flakes are clearly visible [34, 35]. In contrast, the CaO-NPs/CPE image (Fig. 4b) indicates a uniform distribution of CaO-NPs within the paste. After the formation of a PoDA coating on the surfaces of BCPE and CaO-NPs/CPE (Fig. 4c and d, respectively), the graphite flakes were no longer observed, confirming successful surface modification [36].

###  Estimation of electroactive surface area

**Fig. 5:**
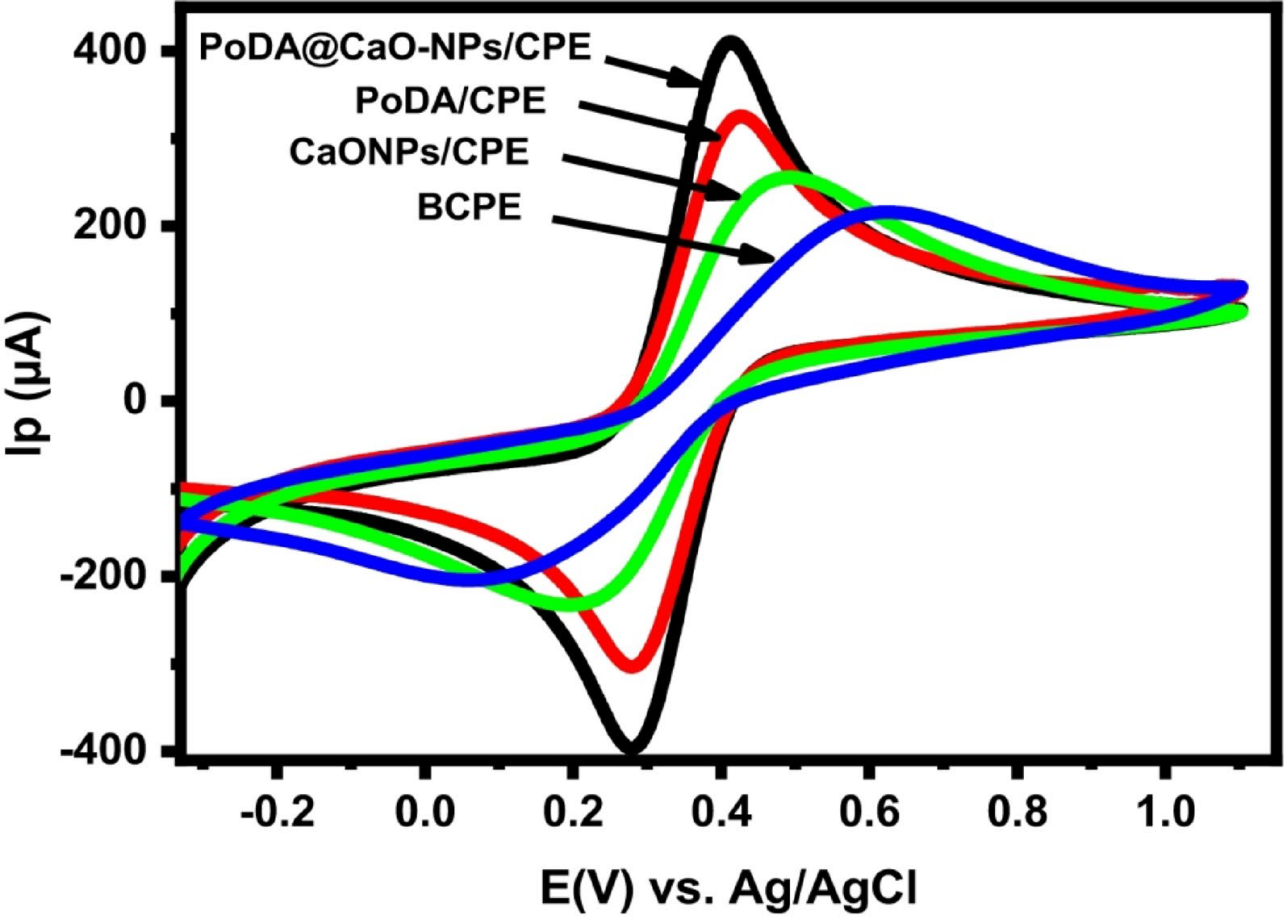
CVs of 1.0 mM [Fe(CN)_6_] ^3–/4–^ in 0.1 M KCl (pH = 7) at BCPE, CaO-NPs/CPE, PoDA/CPE, and PoDA@CaO-NPs/CPE

Cyclic voltammetry (CV) of 1 mM [Fe(CN)₆]³⁻/⁴⁻ in 0.1 M KCl was performed on the working electrodes to estimate their electroactive surface area. As shown in Fig. 5, BCPE exhibited the lowest current response, while PoDA@CaO-NPs/CPE displayed the highest current compared with PoDA/CPE and CaO-NPs/CPE. The peak-to-peak separation potential (ΔEp) of PoDA@CaO-NPs/CPE was 0.46 V lower than that of BCPE, indicating that the incorporation of CaO-NPs and the PoDA film enhanced electron transfer between [Fe(CN)₆]³⁻/⁴⁻ and the electrode surface. Consequently, the conductivity of PoDA@CaO-NPs/CPE was significantly improved. Using the Randles–Ševčík equation (Eq. 1) [37], the electroactive surface area (A) of the fabricated electrodes was calculated .

In the Randles–Ševčík equation, Ip represents the peak current, n is the number of electrons transferred in the electrochemical reaction (equal to 1), ν is the scan rate (mV/s), D is the diffusion coefficient of [Fe(CN)₆]³⁻/⁴⁻ (7.60 × 10⁻⁶ cm²·s⁻¹), and C₀ is the concentration of [Fe(CN)₆]³⁻/⁴⁻ in mol·cm⁻³. Based on the calculations, the electroactive surface areas (A) of BCPE, CaO-NPs/CPE, PoDA/CPE, and PoDA@CaO-NPs/CPE were 0.0165, 0.0486, 0.0530, and 0.0864 cm², respectively.Compared with BCPE, the electroactive surface areas of CaO-NPs/CPE and PoDA/CPE were approximately 2.95-fold and 3.2-fold higher, respectively, confirming the enhanced electrochemical properties of CaO-NPs and PoDA. Notably, PoDA@CaO-NPs/CPE exhibited an electroactive surface area about 5.2 times larger than that of BCPE, highlighting the synergistic effect of CaO-NPs and PoDA in boosting the electrocatalytic activity of the electrode .

### Electrochemical behavior of LNZO using CV

**Fig. 6:**
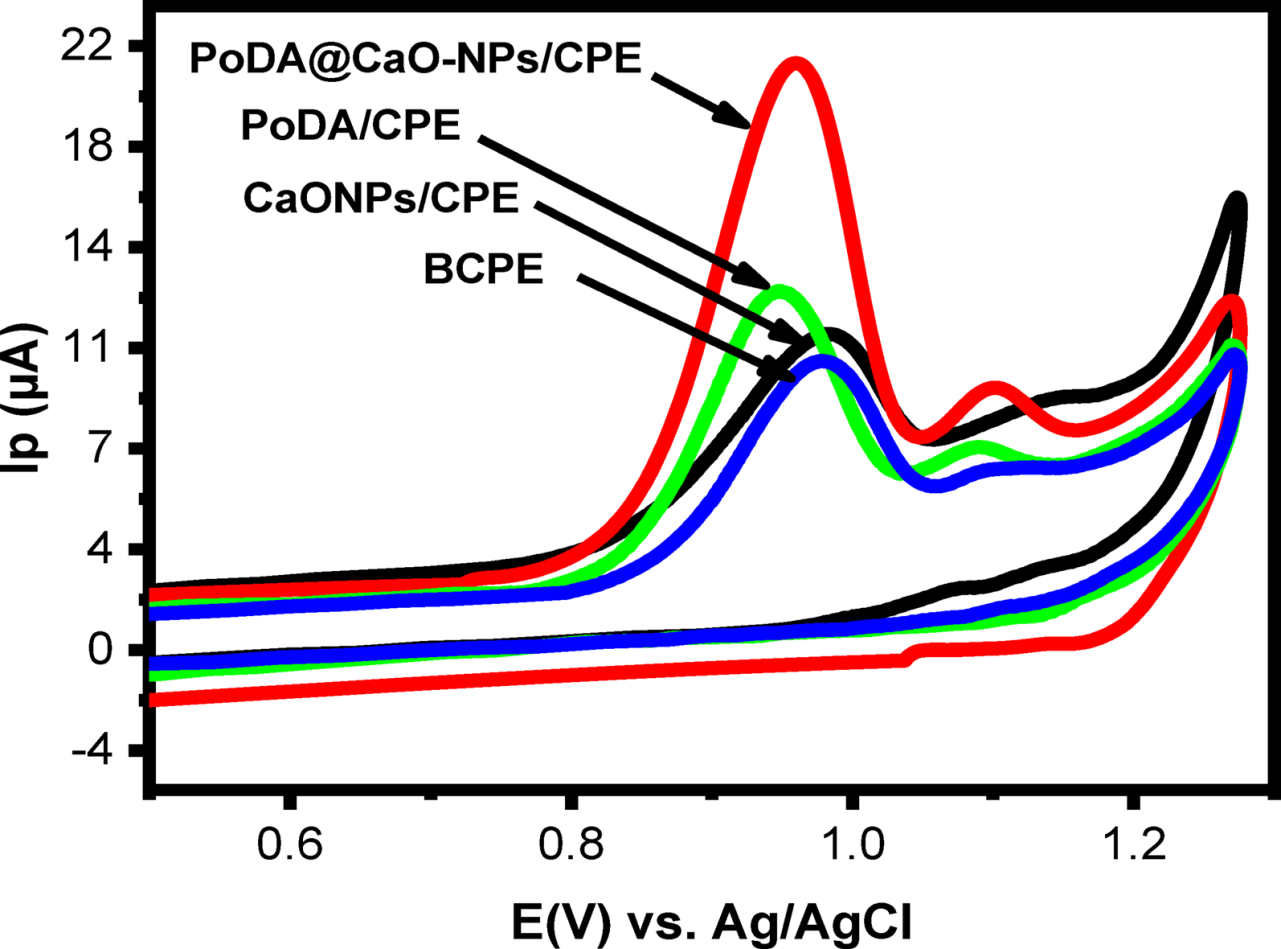
CVs at various electrodes utilizing a scan rate of 50 mV/s in the presence of PBS (pH = 3.0) including LNZO (50 µM)

CV was performed in the potential range of 0.5–1.3 V at a scan rate of 50 mV/s to investigate the electrochemical behavior of 50 µM LNZO at BCPE, CaO-NPs/CPE, PoDA/CPE, and PoDA@CaO-NPs/CPE in PBS (pH 3.0). As shown in Fig. 6, the peak potential (Ep) of BCPE and CaO-NPs/CPE was approximately 0.97 V, while that of PoDA/CPE and PoDA@CaO-NPs/CPE shifted slightly to 0.95 V and 0.96 V, respectively. Among the tested electrodes, PoDA@CaO-NPs/CPE exhibited the highest current response (20.97 µA), compared with 10.32 µA for BCPE, 11.28 µA for CaO-NPs/CPE, and 12.82 µA for PoDA/CPE.

This enhancement can be attributed to the synergistic effect of CaO-NPs and PoDA, which provide a large surface area, high adsorption capacity, and improved electron mobility [31, 38–40]. These features facilitate the accumulation of LNZO molecules at the electrode surface, thereby promoting its electro-oxidation. Furthermore, the disappearance of the reduction peak confirms the irreversible nature of the electrochemical process of LNZO.

### Effect of the different parameters on LNZO peak current, including number of electro-polymerization cycles, supporting electrolyte, pH solution, and scan rate was investigated

**Fig. 7:**
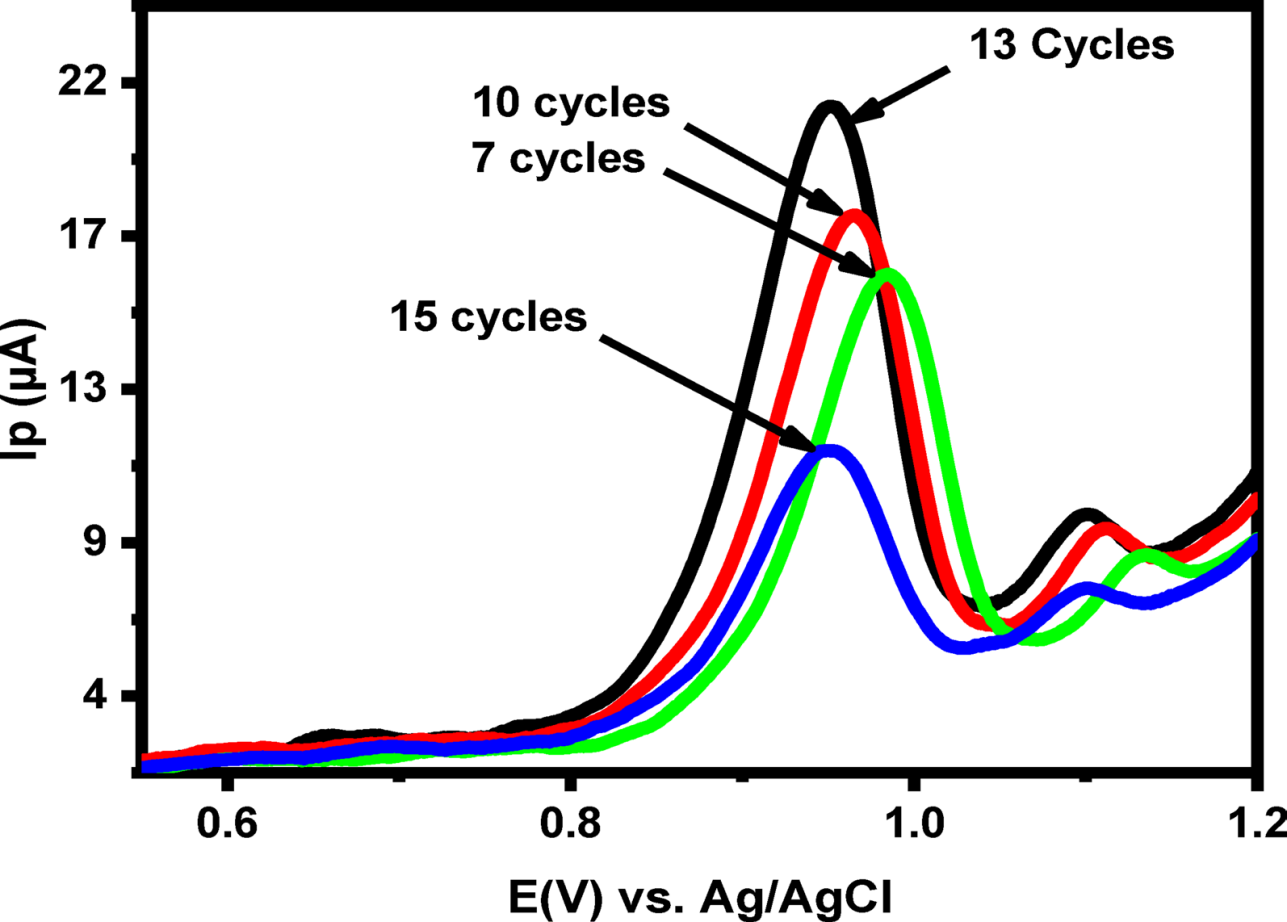
LSVs of LNZO (50 µM) at 7.0, 10.0, 13.0, and 15.0 cycles of electro-polymerization at PoDA@CaO-NPs/CPE in PBS (pH = 3.0) at 50 mV/s scan rate

The current response of LNZO was strongly influenced by the number of electropolymerization cycles of D-alanine (PoDA) applied at BCPE. As shown in Fig. 7, the anodic current increased steadily from 7 to 13 cycles and then decreased when the number of cycles exceeded 13. This decline is attributed to the formation of a thicker PoDA film, which hinders electron transfer across the electrode surface. Therefore, the optimum number of electropolymerization cycles was determined to be 13 .

**Fig. 8:**
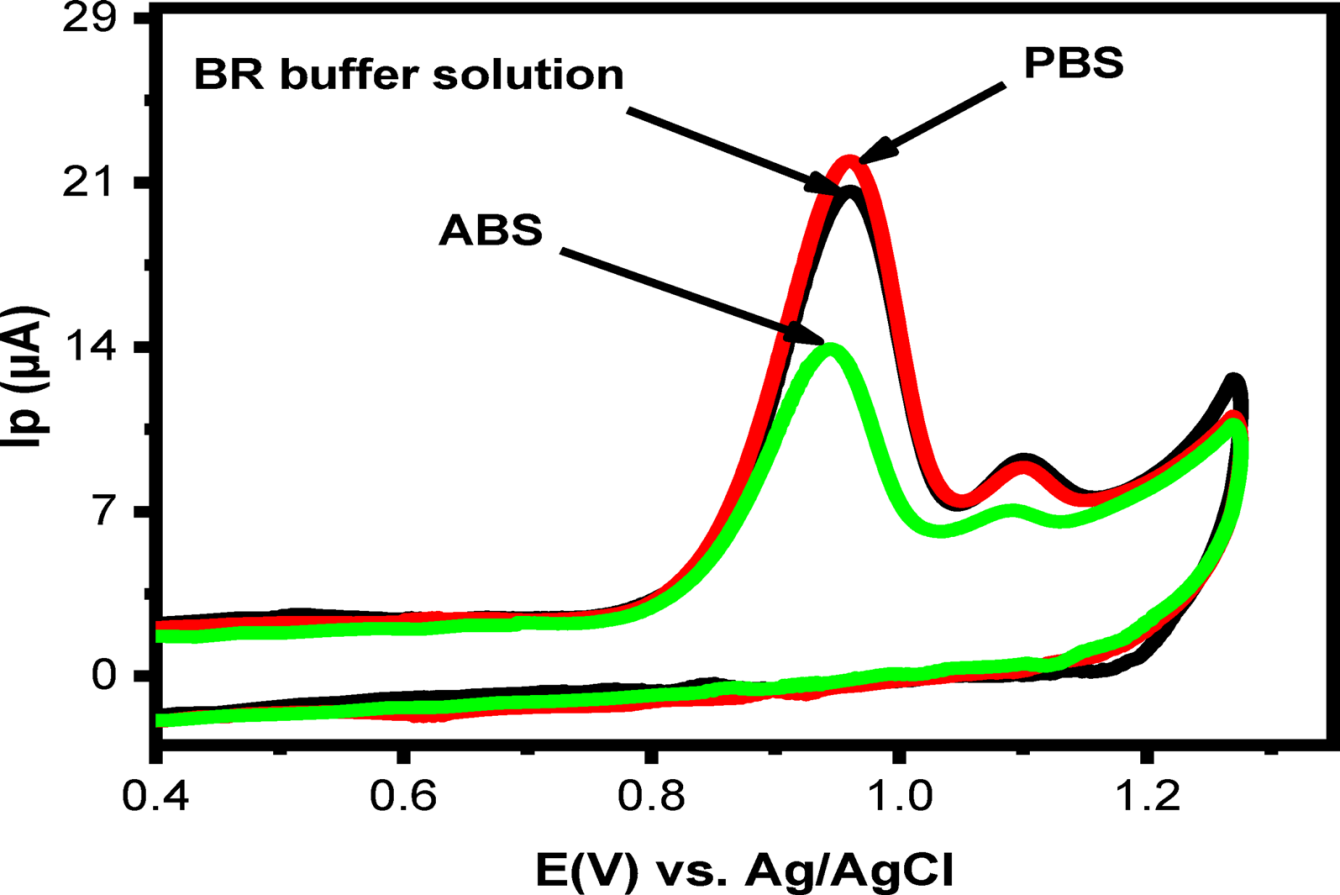
CVs of 50 µM LNZO at 50 mV/s sweep rate containing various supporting electrolytes at PoDA@CaO-NPs/CPE

The electrochemical behavior of LNZO is strongly influenced by the type of supporting electrolyte used. Therefore, selecting the electrolyte that yields the highest current response and the most well-defined anodic peak is essential. As shown in Fig. 8, the effect of different electrolytes—including phosphate buffer solution (PBS), Britton–Robinson (BR) buffer, and acetate buffer solution (ABS)—on the oxidation peak of 50 µM LNZO was evaluated under identical conditions. Although all three electrolytes produced sharp oxidation peaks, the maximum peak current was obtained in PBS. Accordingly, PBS was selected as the optimal supporting electrolyte for subsequent experiments .

**Fig. 9:**
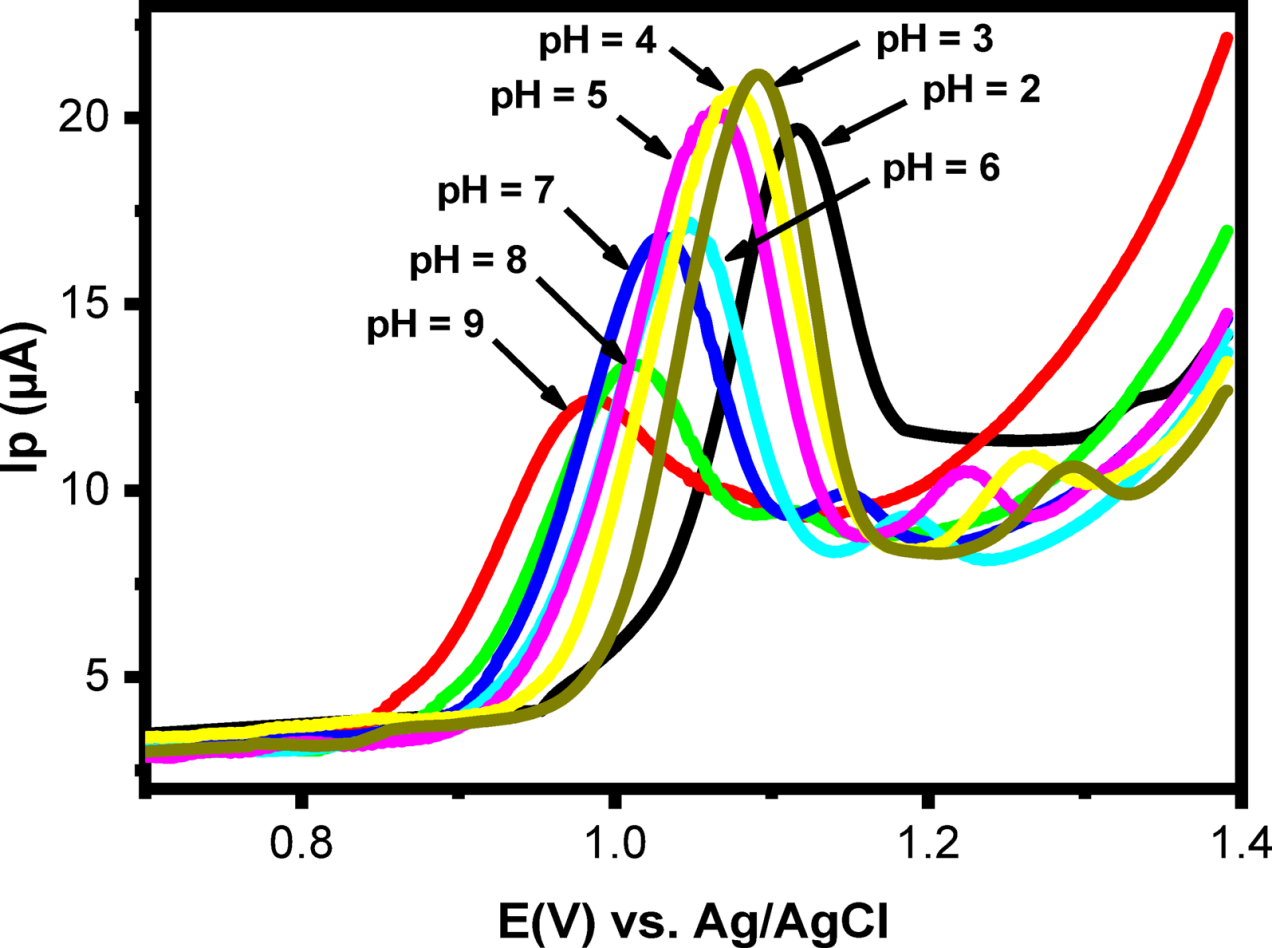
LSVs of 50 µM LNZO at PoDA@CaO-NPs/CPE in PBS at 50 mV/s sweep rate at pH values from 2.0 to 9.0

Since the electrochemical behavior of an analyte can be strongly influenced by the pH of the supporting electrolyte, optimizing the pH of PBS was essential. Linear sweep voltammetry (LSV) was used to study the effect of pH (2.0–9.0) on the anodic peak current of 50 µM LNZO, as shown in Fig. 9. The maximum anodic peak current was observed at pH 3.0, indicating the strongest electrocatalytic activity of PoDA@CaO-NPs/CPE toward LNZO. Above this value, the current intensity gradually decreased.

At lower pH values, LNZO exhibited two distinct oxidation peaks, which merged into a single peak at higher (basic) pH values. This phenomenon is likely related to prototropic changes in the LNZO molecule rather than alterations in its interaction with PoDA@CaO-NPs/CPE [20]. Accordingly, pH 3.0 was selected as the optimal condition, consistent with previous reports [22].

An increase in pH also caused a progressive shift of the oxidation potential (Ep) toward less positive values, demonstrating that the electro-oxidation of LNZO is proton-assisted. A linear relationship between Ep and pH was obtained (Eq. 1).

1 $${\mathbf{Ep}}=1.15 - 0.026~{\mathbf{pH}}~~~~~~~~~~\left( {{{\mathbf{r}}^{2~}}=0.998} \right)~$$

The slope of − 0.026 V/pH indicates that the number of electrons participating in the oxidation of LNZO is approximately twice the number of protons, consistent with the theoretical Nernst slope of − 0.059 V/pH [20].

**Fig. 10:**
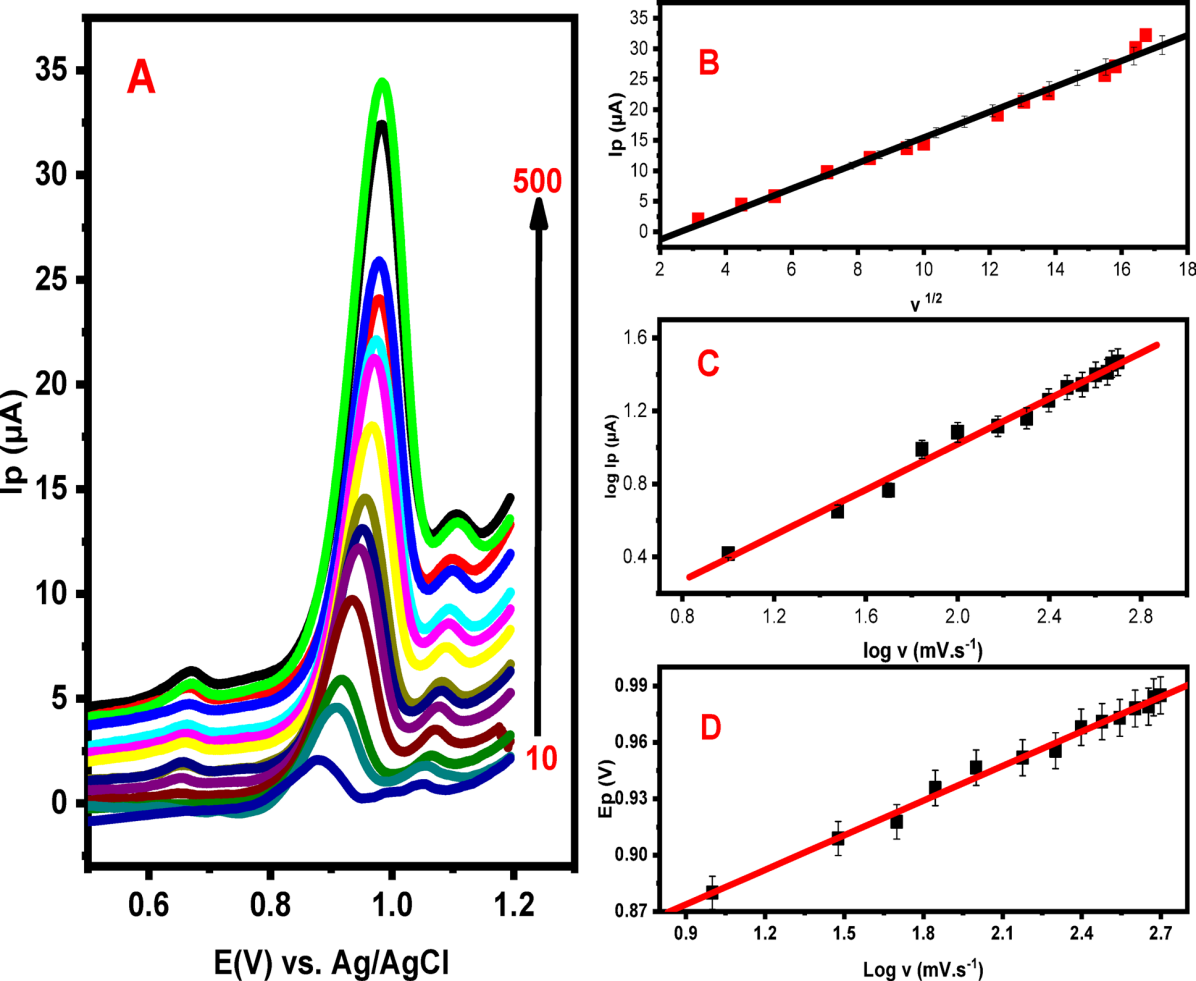
**A** LSVs of LNZO (10 µM) in PBS (pH = 3.0) at scan rates of 10, 30, 50, 70, 100, 150, 200, 250, 300, 350, 400, 450, 470, and 500 mV/s at PoDA@CaO-NPs/CPE. **B** The Plot of Ip of 10 µM LNZO versus √$\nu$ in PBS (pH = 3.0) at PoDA@CaO-NPs/CPE. **C** The plot of log Ip of LNZO (10 µM) against log $\nu$ in PBS (pH = 3.0) at PoDA@CaO-NPs/CPE. **D** The plot of Ep Vs. log $\nu$ at PoDA@CaO-NPs/CPE

The effect of scan rate on the electrochemical response of LNZO was examined using LSV to gain insight into the kinetics of the PoDA@CaO-NPs/CPE process. Measurements were performed in PBS (pH 3.0) containing 10 µM LNZO at scan rates ranging from 10 to 350 mV/s (Fig. 10A). As the scan rate increased, the anodic peak current increased proportionally. A slight positive shift in Ep was also observed, indicating that the electro-oxidation of LNZO at PoDA@CaO-NPs/CPE is an irreversible process [41].

A linear relationship was obtained between the peak current (Ip) of LNZO and the square root of the scan rate (√ν), as shown in Fig. 10B (Eq. 2).

2 $${\mathbf{Ip}}= - 5.46+2.09~\surd {\text{\varvec{\upnu}}}~~~~~\left( {{{\mathbf{r}}^{2~}}=0.994} \right)$$

The electro-oxidation of LNZO was confirmed to be diffusion-controlled, as indicated by the correlation coefficient (*r* = 0.994), which is very close to the theoretical value [24]. Furthermore, as shown in Fig. 10C, a linear correlation was obtained between log Ip and log ν, consistent with regression Eq. (3)

3 $${\mathbf{Log}}~\left( {{\mathbf{Ip}}} \right)= - 0.23+0.62~{\mathbf{log\nu }}~~~\left( {{{\mathbf{r}}^{2~}}=0.993} \right)$$

The oxidation of LNZO was further confirmed to follow a diffusion-controlled mechanism, as evidenced by the slope of the corresponding equation (α = 0.62), which is close to the theoretical value of 0.50 [42]. Similarly, Eq. (4) describes the linear relationship between the oxidation potential (Ep) of LNZO and log ν, as illustrated in Fig. 10D .

4 $${\mathbf{Ep}}~\left( {\mathbf{V}} \right)=0.82+0.061~{\mathbf{log\nu }}~~~~~~~~~~\left( {{{\mathbf{r}}^{2~}}=0.996} \right)$$

**Scheme 1:**
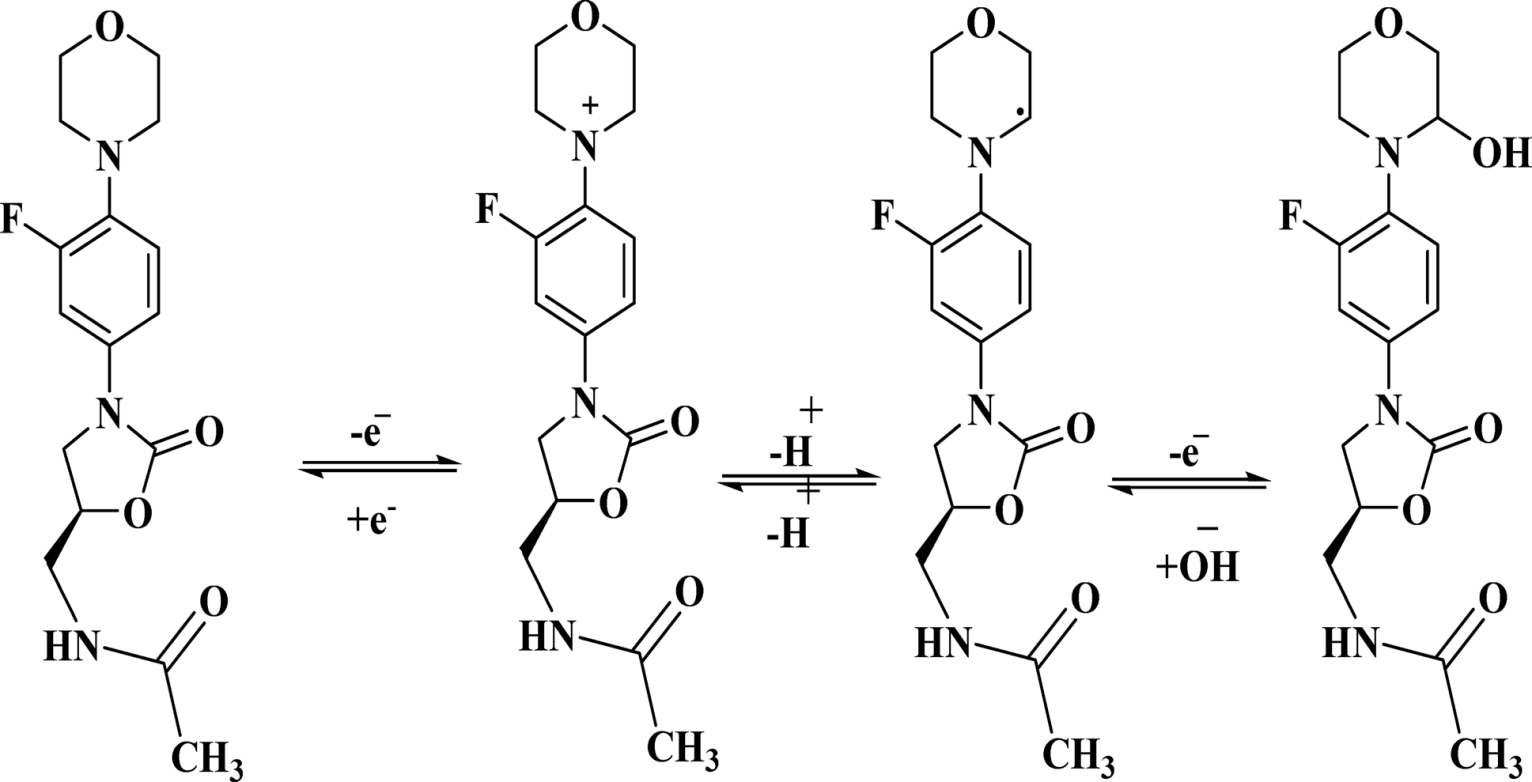
Proposed electrochemical oxidation process of LNZO

According to Laviron’s theory [43], this relationship provides a slope of 0.059/αn. From the calculated value of n (1.6), it can be inferred that the electro-oxidation of LNZO involves the transfer of two electrons. Thus, the proposed oxidation mechanism of LNZO is consistent with previous reports (Scheme 1) [21] :

### Analytical performance of PoDA@CaO-NPs/CPE

**Fig. 11:**
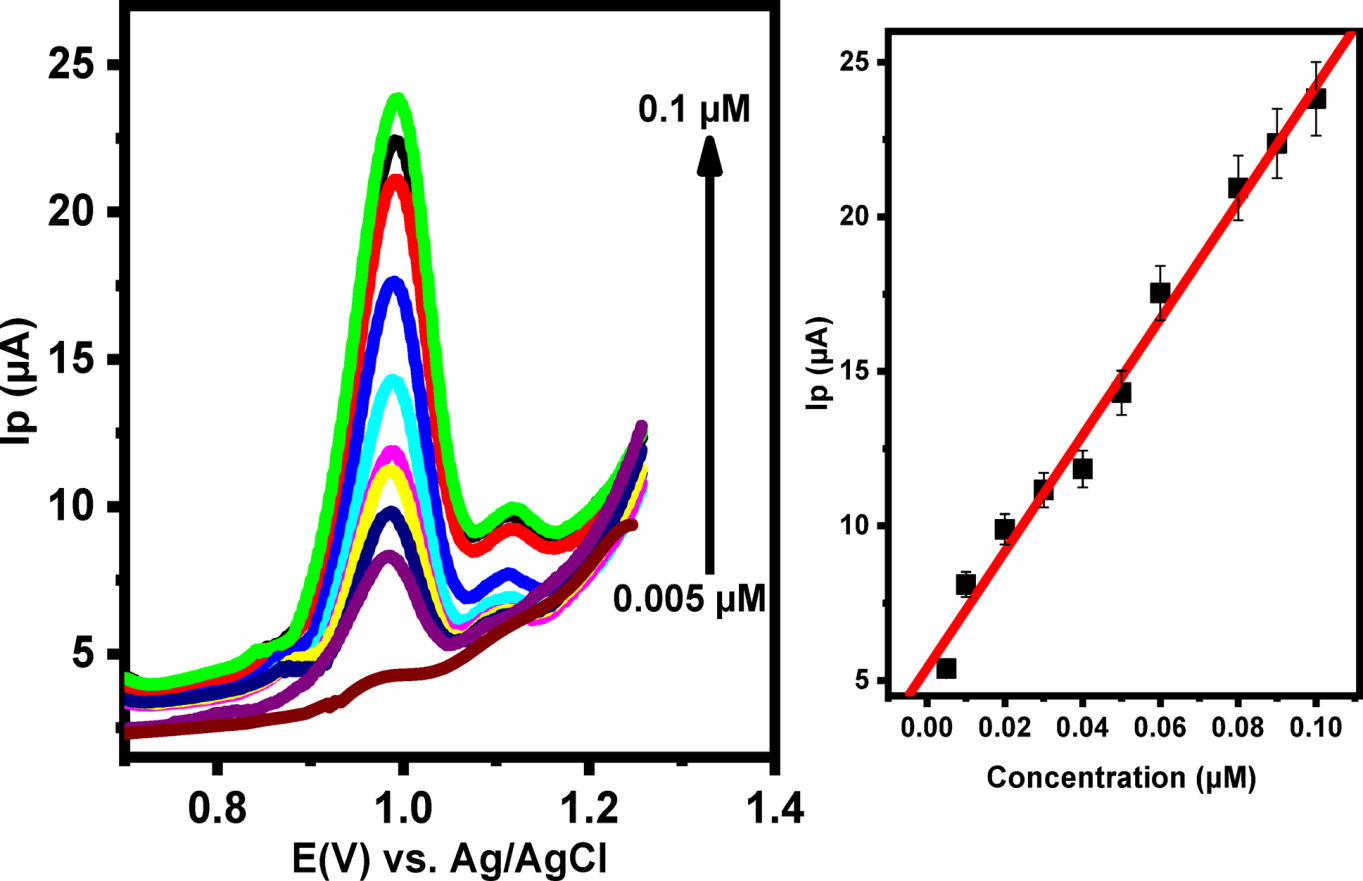
**A** DPVs for quantification of LNZO concentrations (0.005, 0.009, 0.01, 0.02, 0.03, 0.04, 0.05, 0.06, 0.08, 0.09 and 0.1µM) in PBS (pH = 3.0) at PoDA@CaO-NPs/CPE (**B**) The calibration curve of Ip Vs. [LNZO] at PoDA@CaO-NPs/CPE

Differential pulse voltammetry (DPV) was employed to quantitatively determine LNZO using PoDA@CaO-NPs/CPE under optimized experimental conditions. This technique significantly enhanced the electrode sensitivity. The DPV parameters were adjusted to obtain a well-defined peak shape, and the optimal values were determined as follows: step width, 0.02 s; step height, 4 mV; pulse height, 40 mV; and pulse width, 0.01 s. As shown in Fig. 11, the anodic peak current increased progressively with LNZO concentrations ranging from 0.005 to 0.1 µM. A strong linear correlation was established, as described below :

5 $${\mathbf{Ip}}~=188.31~{\mathbf{C}}+5.39~~~~~~~\left( {{\mathbf{n}}=11,~{{\mathbf{r}}^{2~}}=0.994~} \right)$$

The limit of detection (LOD) and limit of quantification (LOQ) for LNZO were calculated as 0.00127 µM and 0.0266 µM, respectively, using the standard relationships described below:

$${\mathbf{LOD}}=3~{\raise0.7ex\hbox{${\mathbf{S}}$} \!\mathord{\left/ {\vphantom {{\mathbf{S}} {{\mathbf{m}}~~~}}}\right.\kern-0pt}\!\lower0.7ex\hbox{${{\mathbf{m}}~~~}$}}{\mathbf{and}}~~{\mathbf{LOQ}}=10{\raise0.7ex\hbox{${~{\mathbf{S}}}$} \!\mathord{\left/ {\vphantom {{~{\mathbf{S}}} {\mathbf{m}}}}\right.\kern-0pt}\!\lower0.7ex\hbox{${\mathbf{m}}$}}$$

In these equations, S represents the standard deviation (SD) of three measurements of the anodic peak current at the lowest LNZO concentration within the linear range, while m denotes the slope of the calibration curve. The very low LOD achieved demonstrates that PoDA@CaO-NPs/CPE can reliably detect trace amounts of LNZO with high precision. A comparison of the electroanalytical performance of PoDA@CaO-NPs/CPE with other modified electrodes for LNZO determination is summarized in Table 1. .

**Table 1:** Comparing PoDA@CaO-NPs/CPE with other modified electrodes that have been already published for LNZO quantifying

Electrode	Technique	Limit of detection (µM)	References
ERGO-BEN/GCE	DPV	0.1	[44]
BDD electrode	SWV	0.15	[45]
GCE	DPV	0.15	[23]
MWCNTs/CPE	SWV	0.0016	[21]
CPE	SWV	0.0093	[18]
PGE	SWV	0.0014	[18]
MWCNTs/BCG/CPE	DPV	0.0076	[19]
PoDA@CaO-NPs/CPE	DPV	0.00127	Present work

ERGO-BEN, BDD, MWCNTs, BCG, GCE, PGE, and SWV mean graphene oxide-bentonite sodium, boron-doped diamond, multiwalled carbon nanotubes, bromocresol green, glassy carbon electrode, pencil graphite, and square wave voltammetry, respectively

### Reproducibility, repeatability, and stability

The reproducibility of the modified sensor was evaluated by performing five consecutive measurements (*n* = 5) of 0.09 µM LNZO using five independently prepared PoDA@CaO-NPs/CPEs. The results showed a relative standard deviation (RSD) of 4.9%, indicating satisfactory reproducibility. Repeatability was assessed by recording eight successive CV scans (*n* = 8) with the same electrode, yielding an RSD of 2.6%.

The stability of the developed sensor was examined by regenerating a PoDA film on the same BCPE after polishing its surface and monitoring the anodic current response of 0.09 µM LNZO daily. After two weeks, the peak current decreased by only 6.85% compared with the initial value, confirming the excellent stability of the proposed electrode .

### Interferencses study

In pharmaceutical tablets and human serum samples, LNZO may coexist with various interfering species, including inorganic ions and organic compounds. Therefore, the effect of potential interferents on LNZO determination (10 µM) in PBS (pH 3.0) at PoDA@CaO-NPs/CPE was evaluated using DPV. As summarized in Table 2, the oxidation peak current of LNZO decreased by only 1.02–3.61% relative to the original signal after the addition of 2500 µM magnesium stearate and starch, 1000 µM arginine, lactose, urea, and L-cystine, and 3500 µM of Mg²⁺, PO₄³⁻, Na⁺, K⁺, Zn²⁺, Fe³⁺, and Cl⁻.

These results demonstrate that the fabricated sensor possesses excellent selectivity for LNZO analysis, even in the presence of common coexisting substances.

**Table 2:** Influence of various metal ions and organic compounds on the peak current signal of 10 µM LNZO in the presence of PBS (pH = 3.0) at PoDA@CaO-NPs/CPE

Interfering agents (A) (3500 µM)	I_*p*.a._ change %	Interfering agents (B) (2500 µM)	I_*p*.a._ change %	Interfering agents (C) (1000 µM)	I_*p*.a._ change %
Mg^2+^	-2.35	Starch	-2.42	Lactose	-2.58
PO_4_^3−^	-1.02	Magnesium stearate	-2.70	L-cystine	-3.26
k^+^	-1.02			Urea	-3.61
Zn^2+^	-2.75			Arginine	-2.01
Fe^2+^	-2.84				
Cl^−^	-1.03				

### Real samples analysis

The practical applicability of PoDA@CaO-NPs/CPE was evaluated by quantifying LNZO in Averozolid™ commercial tablets and human serum samples using the standard addition method. Known amounts of LNZO were spiked into the prepared real samples, and the concentrations were determined by DPV. The recovery values were calculated by comparing the measured concentrations with the initially added amounts (Table 3).

The recoveries ranged from 95.80% to 100.20%, demonstrating the high efficiency of PoDA@CaO-NPs/CPE. These results confirm that the proposed sensor is highly suitable for real sample analysis in complex matrices .

**Table 3:** Practical application of PoDA@CaO-NPs/CPE for measuring LNZO concentration in Averozolid™ tablet and human serum samples

Sample	LNZO added (µM)	LNZO found (µM)	Recovery %
Averozolid™ (600 mg)	5.0	4.92 ± 0.08	98.40
	10.0	10.02 ± 0.02	100.2
	15.0	14.98 ± 0.02	99.87
Human serum	5.0	4.85 ± 0.14	97.0
	10.0	9.58 ± 0.40	95.80
	15.0	14.95 ± 0.05	99.67

*S.D represents the standard deviation from three measurements

## Conclusion

Voltammetric sensors play a vital role in detecting various analytes, which is essential for quality assessment in food, pharmaceuticals, biological fluids, and environmental samples [46–50]. In this work, we demonstrated the successful application of a carbon paste electrode (CPE) modified with calcium oxide nanoparticles (CaO-NPs) and electropolymerized D-alanine (PoDA) for the quantification of LNZO. The proposed PoDA@CaO-NPs/CPE provided a wide linear detection range (0.005–0.1 µM), along with excellent selectivity in the presence of potential interfering species.

The developed electrode also exhibited satisfactory stability, repeatability, and reproducibility, with relative standard deviations (RSD) of 6.85%, 2.6%, and 4.9%, respectively. Importantly, the sensor demonstrated high efficiency in real sample analysis, achieving recovery values between 95.70% and 100.20%. These findings confirm that PoDA@CaO-NPs/CPE is an effective, reliable, and practical tool for LNZO detection in pharmaceutical and biological samples .

## Supplementary Information

Supplementary material 1.

## Data Availability

The datasets generated and/or analyzed during the current study are available from the corresponding author upon reasonable request.
