# Supplementary material for: A novel low –cost and sensitive sensor for the voltammetric nano detection of linezolid antibiotic in real samples using carbon paste electrode modified with calcium oxide nanoparticle combined with electropolymerized D-alanine
Source: BMC Chem. 2025 Nov 15;19(1):305. doi: 10.1186/s13065-025-01663-3 (PMC12619255; doi:10.1186/s13065-025-01663-3)
Supplement: Supplementary file 1 — Supplementary material 1. [file 13065_2025_1663_MOESM1_ESM.docx]

Supplementary material

A bar chart comparing current responses of 10 µM LNZO in the presence of interferences.
